# Unveiling the role of agricultural insurance in driving rural industry revitalization in China

**DOI:** 10.1016/j.heliyon.2024.e34483

**Published:** 2024-07-11

**Authors:** Dainan Hou, Xin Wang

**Affiliations:** a School of Business, Minnan Normal University, Zhangzhou, China; b College of Life Science, Longyan University, Longyan, China; c Chinese International College, Dhurakij Pundit University, Bangkok, Thailand

**Keywords:** Agricultural insurance, Rural industries, Functional decomposition, Bidirectional fixed effect, China

## Abstract

This study aims to investigate the influence of agricultural insurance on the development of the agricultural industry. Employing the functional decomposition approach in systems engineering, we analyze the mechanisms by which agricultural insurance affects the development of rural industries. Additionally, we conduct an empirical analysis using panel data from Chinese provinces between 2011 and 2019, employing a two-way fixed effects model. The findings reveal a substantial and positive impact of agricultural insurance on the development of rural industries. Furthermore, we identify regional heterogeneity in the influence of agricultural insurance on rural industry development. Specifically, in major grain-producing provinces, the effect of agricultural insurance on rural industry development is not statistically significant. In contrast, non-grain-producing provinces experience a significantly positive impact on rural industry development from agricultural insurance. The outcomes of this study offer valuable insights for policymakers to promote rural industry development through the effective utilization of agricultural insurance.

## 1 Introduction

The 19th National Congress of the Communist Party of China (CPC) first proposed prioritizing the development of agriculture and rural areas, implemented the rural revitalization strategy, and aligned it with the seven major tactics for realizing the objective of a moderately prosperous society [1]. Consequently, the central government issued vital policy documents and laws and regulations related to rural revitalization, such as the “Opinions of the Central Committee of the CPC and the State Council on the Implementation of the Rural Revitalization Strategy,” the “Rural Revitalization Strategy Plan (2018–2022)," and the “Rural Revitalization Promotion Law.” These policies have established development directions and legal protection for rural revitalization. Furthermore, the 20th National Congress of the CPC, held on October 16, 2022, advocated an all-out promotion of rural revitalization, making profound statements and comprehensive provisions to foster rural revitalization [2]. The revitalization of rural industries is the bedrock for rural revitalization. For rural areas to thrive, their industries must also thrive. Enhancing the quality and efficiency of industrial development and boosting endogenous motivation are crucial to rural revitalization, as they are directly linked to agricultural development and the increase in farmers' income [3].

Agricultural insurance plays a pivotal role within the rural financial system as a vital tool for mitigating agricultural risks and is extensively utilized by countries worldwide [4]. Given its quasi-public good nature, agricultural insurance requires partial government support. In 2007, China implemented a central government subsidy policy for agricultural insurance premiums, leading to rapid growth in the Chinese agricultural insurance market. In 2022, the premium scale of agricultural insurance reached 121.9 billion yuan, providing risk protection valued at 5.46 trillion yuan for 167 million households [5]. Recognizing its significant contribution to the rural revitalization strategy, the “Plan for Rural Revitalization (2018–2022)" explicitly includes agricultural insurance in the section on “Improving the Agricultural Support and Protection System.” By analyzing the impact of agricultural insurance on rural industries, we can examine the supportive effect of agricultural insurance on rural revitalization and evaluate the alignment of the current agricultural insurance policy with agricultural advancements. This analysis forms the basis for optimizing agricultural insurance.

In the existing literature, research on the impact of agricultural insurance on rural industries primarily concentrates on the effects of agricultural insurance on agriculture. Specifically, it examines the following two aspects.

The first aspect of the research focuses on the impact of agricultural insurance on agricultural output. Three predominant viewpoints exist regarding this impact. According to the first viewpoint, agricultural insurance exerts a positive influence on agricultural output [6,7] and can enhance agricultural production and efficiency [8]. Conversely, the second viewpoint argues that agricultural insurance hinders climate change adaptation in agriculture. Crop insurance has a negative effect on average yields under climate change and alters the warming effect on yields over time [9]. The third viewpoint suggests a U-shaped relationship between agricultural insurance and agricultural production. Ren et al. [10] propose that the level of agricultural insurance coverage can enhance agricultural production efficiency among farmers up to a specific threshold level. However, once the coverage level surpasses the threshold for moral hazard, agricultural insurance will impede the efficiency of agricultural production.

The second aspect of this research examines the effect of agricultural insurance on the utilization of agricultural chemicals. Scholars have identified that agricultural insurance can potentially influence the usage of chemicals, leading to two contradictory viewpoints: one suggests that agricultural insurance may decrease the utilization of chemicals such as fertilizers and pesticides [[11], [12], [13], [14]], while the other proposes that it may actually increase their usage [15]. Advancing research in these two areas is of significant theoretical value and has important policy implications for analyzing the impact of agricultural insurance on the agricultural sector. However, due to the comprehensive promotion of China's rural revitalization strategy and the further development of its rural industries, there has been a growing focus on the role of agricultural insurance in these sectors. Therefore, this paper specifically concentrates on China as the research subject, explores the theoretical mechanisms through which agricultural insurance influences rural industry development, and empirically tests these mechanisms.

In comparison to existing literature [3,[5], [6], [7], [8], [9], [10], [11], [12], [13], [14]], this paper makes three significant contributions: a problem-solving approach [3,5,6], careful selection of empirical variables [10,13,14], and exploration of regional heterogeneity [13].

(1) Regarding the problem-solving approach [3,5,6], agricultural insurance is widely recognized as a crucial tool for diversifying agricultural risks in many countries. Previous studies have highlighted the various functions of agricultural insurance. To analyze the impact of each function on the development of rural industries, this paper adopts the functional decomposition method. The aim is to establish a solid theoretical foundation for subsequent empirical analysis. This methodological approach is innovative and contributes to the existing literature on agricultural insurance.
(2) In terms of the selection of empirical variables [10,13,14], this paper takes into account the potential time-lag effect of agricultural insurance compensation on farmers' production and livelihood in the following period. Consequently, the baseline regression employs the lagged agricultural insurance compensation per acre as the explanatory variable. To examine the stability of the results, the current agricultural insurance premium income is used as the explanatory variable in a separate test. By considering both lagged compensation and current premium income, the paper ensures the robustness and reliability of the empirical findings.
(3) In contrast to previous literature [13], this study deviates from the conventional regional divisions based on north-south or east-west directions. Instead, China's provinces are categorized into two distinct groups based on their grain production: major grain-producing provinces and non-major grain-producing provinces. This novel classification allows for a comprehensive analysis of the varying roles played by agricultural insurance in promoting rural industry development within different regions. Consequently, the paper provides substantial evidence to support the tailored recommendations presented in the subsequent sections. The objectives of this study are to examine the impact of agricultural insurance on rural industrial development from the perspective of agricultural insurance functions, and to examine the issue of regional heterogeneity.

The remaining sections of this paper are structured as follows: Section 2 presents a comprehensive review of the literature on the correlation between agricultural insurance and both rural income and agricultural production. Section 3 utilizes the functional decomposition methodology to evaluate the influence of agricultural insurance on the development of rural industries, while also putting forward a set of research hypotheses. Section 4 offers an outline of the research design, including the empirical model, data sources, and variable selection. Section 5 focuses on the empirical analysis and features the computation of the rural industry development index, benchmark regression, heterogeneity analysis, and robustness checks. In Section 6, the research findings are analyzed and compared to prior studies, followed by the proposal of development recommendations. Finally, Section 7 concludes the paper by discussing its drawbacks and future prospects.

## 2 Literature review

This article undertakes a comprehensive literature review, focusing on two perspectives: the relationship between agricultural insurance and farmers' income, as well as agricultural production. This approach aligns with the central theme of the research.

### 2.1 Agricultural insurance and farmers' income

In the context of agricultural insurance contracts, farmers assume dual roles as both the insured and the beneficiaries. Consequently, the impact of agricultural insurance on farmer income has garnered significant attention from scholars. However, a consensus regarding the effect of agricultural insurance on farmer income has yet to be reached. While some studies, such as those conducted by Skees et al. [16] on impoverished farmers in Mexico, Kshetri [17] on small farmers in developing countries, and Agbenyo on farmers in Ghana, have identified a positive relationship between agricultural insurance and farmer income, empirical evidence from Huang et al. [18] suggests a small effect. Conversely, other studies have reached different conclusions [19]. Fusco et al. [20] found a negative effect of agricultural insurance on farmers' net income within the context of Italian agricultural insurance, attributing it to a corresponding increase in premiums as farmers' income rises. In a survey conducted by McIntosh et al. [21] among Ethiopian farmers, it was discovered that farmers with high marginal returns on inputs expressed willingness to purchase insurance, but only those with low marginal returns followed through with their intention. Furthermore, the extent and scope of insurance coverage were identified as crucial factors shaping the role of agricultural insurance on farmers' operating net income, as demonstrated by Liu et al. [22]. The study revealed that an increase in government premium subsidies prior to agricultural disasters strengthens the level of protection provided by agricultural insurance and stimulates the breadth and depth of coverage, which ultimately influences farmers' operating net income. Conversely, following a disaster, compensatory benefits derived from coverage have been found to hinder the positive impact of coverage on farmer income.

### 2.2 Agricultural insurance and agricultural production

An analysis of the impact of agricultural insurance on farmers' production behavior can offer valuable insights into its influence on agricultural output. Initially, academic research has primarily focused on examining the effects of agricultural insurance on agricultural investment and the scale of planting and breeding. Innes [23] argues from an economic and political standpoint that the absence of government policies supported by income insurance, implemented post-crisis, can lead to farmers' excessive participation in agricultural production, ultimately resulting in inadequate output. Similarly, Glauber et al. [24] have observed significant growth in crop insurance programs; however, crop insurance plans have not replaced other forms of disaster relief programs as the sole source of aid. On the other hand, Roll et al. [8] have demonstrated the effectiveness of salmon farming insurance in enhancing production and efficiency while reducing capital and labor inputs. Additionally, Kim et al. [25] have found that crop insurance can reduce farm divestment and exit decisions. Secondly, research has examined the impact of agricultural insurance on the use of agricultural chemicals, which remains a subject of debate in academia. While some scholars believe that agricultural insurance might lead to an increase in chemical usage [15,26,27], Fang et al. [13] have discovered that crop insurance has a positive influence on green total factor productivity in agriculture. Furthermore, additional research has indicated that agricultural insurance can also contribute to a decrease in chemical usage, contrary to the beliefs of certain scholars [11,28,29]. Lastly, research has been conducted to understand the influence of agricultural insurance on the adoption of agricultural technology. Salazar et al. [30] have observed a negative association between participation in crop insurance and the adoption of modern irrigation technology. In contrast to their findings, Sellars et al. [31] have recently demonstrated that crop insurance has minimal effect on the adoption of drainage water recycling technology.

The existing body of literature has amassed a wealth of research findings on the impact of agricultural insurance on farmers' income and agricultural production. These studies have provided extensive theoretical foundations and empirical methods for further research in this field. Building upon this existing knowledge, the objective of this paper is to enhance and refine our understanding in two key areas. Firstly, we aim to broaden our research perspective beyond solely examining agricultural production and farmers. Specifically, this paper will delve into the role of agricultural insurance in facilitating rural industrial development. By doing so, we aim to provide theoretical support and innovative ideas for the effective utilization of agricultural insurance in promoting sustainable rural revitalization. Secondly, we seek to strengthen our theoretical exploration by introducing the concept of functional decomposition derived from systems engineering theory. This theoretical framework will enable us to systematically analyze and understand the mechanisms through which agricultural insurance influences rural industrial development. By employing this approach, we aim to enhance our comprehension of the intricate relationship between agricultural insurance and the broader context of rural development. By combining these two aspects - expanding our research perspective and employing a theoretical framework rooted in systems engineering theory - we aim to advance the knowledge and understanding of the impact of agricultural insurance on rural industrial development.

## 3 Theoretical analysis

### 3.1 Functional decomposition of agricultural insurance - based on functional decomposition approach

**Fig. 1:**
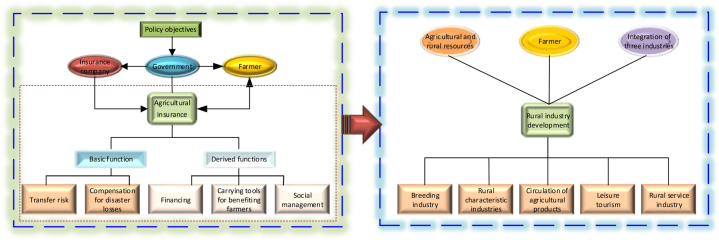
Mechanisms of the impact of agricultural insurance on the development of rural industries.

Functional decomposition plays a critical role in the functional analysis method, which is often employed as an independent approach in relevant literature. The functional analysis method, also known as the functional matrix method, serves as the primary approach for investigating functional solutions [32]. It entails breaking down the overall function of a complex system into individual functional units, which are then solved independently. Subsequently, these individual solutions are combined to generate multiple system solutions, allowing for analysis and comparison in order to identify the optimal functional solution. The process of functional decomposition is essential within the functional analysis method, as it involves breaking down functions into a hierarchical functional tree structure for further analysis [33]. In this study, we adopt the concept of functional decomposition from systems engineering to analyze agricultural insurance. Specifically, we perceive agricultural insurance as a system comprising distinct functions, and decompose its overall function into a series of functional units, which are represented by the red dashed line in Fig. 1.

### 3.2 The impact mechanism and hypothesis of agricultural insurance on the development of rural industries

Rural industries, which are built upon agricultural and rural resources and predominantly driven by farmers, constitute the foundation for rural revitalization. To achieve such revitalization, it is crucial to integrate the primary, secondary, and tertiary sectors by fostering the development of agriculture, rural specialty industries, agricultural product circulation, leisure tourism, and rural services. Agricultural insurance stands out as one of the key risk transfer measures adopted globally, with the Chinese government actively promoting its inclusion in policy objectives. The government encourages farmers to utilize agricultural insurance as a means to transfer agricultural risks and provides them with premium subsidies to strengthen their risk mitigation capabilities. Additionally, the government establishes guidelines and regulations to ensure the sound operation of the agricultural insurance market. The primary function of agricultural insurance is to transfer risks and provide compensation for losses caused by disasters. Moreover, it serves secondary purposes such as capital financing, supporting agricultural benefits, and contributing to social management. As a tool for revitalizing rural industries, agricultural insurance plays a significant role in transferring production risks associated with farming and animal husbandry. It also helps to stimulate rural financial markets, support the implementation of government agricultural policies, and make a positive impact on the revitalization of rural industries.

(1) The Basic Functions of Agricultural Insurance and Rural Industry Revitalization

Given the susceptibility of the agricultural sector to natural and socio-economic uncertainties, along with the unique characteristics of its production process, it is considered a high-risk industry. Agricultural insurance plays a fundamental role in functions such as risk transfer and compensation for losses resulting from disasters. As a result, it enables farmers to transfer natural and market risks [34], mitigating income uncertainty, promoting income equality among farmers [35], sustaining enthusiasm for agricultural production, and fostering the sustainability of agricultural production. Within the agricultural industry, particularly in the domain of crop and livestock farming, the role of agricultural insurance is crucial. This is primarily due to the significance of agricultural products derived from crop and livestock farming, which provide essential raw materials and serve as the foundation for the development of rural characteristic industries, agricultural product distribution, and leisure tourism.

(2) The Derivative Function of Agricultural Insurance and the Revitalization of Rural Industries

Agricultural insurance resembles conventional property insurance in its utilization of collected premiums to establish a large-scale insurance fund. These funds can be temporarily invested by the insurance company in the social reproduction process or the financial market to generate investment income [36]. By supporting agricultural growth and raising funds for the agricultural sector, these insurance funds can serve as a valuable resource. In December 2015, the China Insurance Regulatory Commission authorized the PICC Group to implement a pilot plan that utilized insurance funds to assist agriculture and small and micro-enterprises. The pilot scale initially started at 5 billion yuan and has since grown to 20 billion yuan by 2021. Moreover, agricultural insurance can enhance the credit rating of farmers who require loans but lack collateral. The “agricultural insurance + agricultural credit” model has demonstrated positive outcomes in specific regions of China. Additionally, agricultural insurance provides the government with tools that can support agriculture and aid in the implementation of governmental policies. Furthermore, agricultural insurance serves a social management function by reducing social turbulence and preserving social stability. For example, in China's poverty alleviation efforts, agricultural insurance has played a significant role [37,38]. Therefore, during the process of rural industrial development, agricultural insurance can play an essential role in industrial financing and the implementation of various farmer-centric policies. The impact of agricultural insurance on rural industrial development may vary across different regions, depending on factors such as variations in farmers' production and management capacity, regional policies, and the operations of insurance companies.

Based on the preceding discussion, this article puts forward the following hypotheses.

H1a Agricultural insurance has a positive impact on the development of rural industries.

H1b Agricultural insurance has a negative impact on the development of rural industries.

H2a The impact of agricultural insurance on the development of rural industries varies across different regions.

H2b The impact of agricultural insurance on the development of rural industries does not vary across different regions.

## 4 Research design

### 4.1 Data sources

The time frame for this study has been restricted to the period from 2011 to 2019 due to the substantial number of missing values in the selected variables prior to 2011, as well as the use of different statistical techniques in some data from 2011 onwards. Moreover, due to significant data gaps in Shanghai and Tibet, this study focuses on 29 provinces and regions in China, excluding Shanghai and Tibet.

The data utilized in this study have been obtained from various sources, including the China Statistical Yearbook (2010–2020), China Insurance Yearbook (2010–2020), and China Rural Statistical Yearbook (2010–2020). However, for the variable “rural residents' fixed investment in service and other service industries,” missing values are present for several provinces. To address this issue, a “regression imputation method” has been employed to fill in the gaps.

### 4.2 Empirical strategies

From both theoretical and empirical perspectives, agricultural insurance may play a role in the development of rural industries. To further explore this issue, the following Benchmark estimation model is proposed:

$$ridli_{it}=\alpha +\beta_{1}L.acp_{it}+\beta_{2}\;\ln\;adl_{it}+\beta_{3}\;\ln\;fls_{it}+\beta_{4}\;\ln\;fs_{it}+\beta_{5}\;\ln\;ul+\lambda_{t}+\mu_{i}+\xi_{it}$$

where ridli represents the level of rural industry development, acp represents the level of agricultural insurance development, adl represents the level of agricultural development, fls represents the level of farmer's livelihood, fs represents the degree of financial support, and ul represents the level of urbanization. The subscript i denotes the region (i = 1, 2, …, 29) and t denotes the time period (t = 2011, 2012, … 2019). α is a constant term, $\beta_{n}$ represents the estimated coefficients of the explanatory variables and control variables (n = 1,2 … 5), and ， $\lambda_{t}\mu_{i}$ represent the individual effects and time effects, respectively, and $\xi_{it}$ is the model residual.

### 4.3 Variable definition and description

(1) Dependent Variable

The primary focus of this study is to examine the level of rural industrial development (ridli), which serves as the dependent variable. Previous studies have commonly relied on variables such as the gross output value or added value of the primary industry [39] and the total factor productivity of agriculture [10] to assess the level of agricultural industry development. However, limiting the measurement of rural industrial development to a single indicator or agricultural production efficiency can lead to biased and inaccurate results. Therefore, this study has adopted an indicator system method [40] to establish an evaluation indicator system for rural industrial development. By utilizing this method, the study has computed the rural industrial development index for each province within the study period, enabling a more comprehensive reflection of the level of rural industrial development.

(2) Core Explanatory Variable

The main explanatory variable in this study is the level of agricultural insurance development (acp). Typically, indicators such as agricultural insurance premium income, compensation expenditure, agricultural insurance density, and compensation rate are commonly used to assess the level of agricultural insurance development. However, this study has chosen to focus on the per-mu agricultural insurance compensation expenditure. This choice acknowledges that disaster compensation can provide financial relief to farmers and contribute to a swift resumption of production. Additionally, the current agricultural insurance compensation may have an impact on agricultural production in the following period. Therefore, this study has included the lagged per-mu agricultural insurance compensation expenditure (L.acp) as the primary explanatory variable.

(3) Control Variables

The factors that influence rural industrial development are multifaceted, and in an effort to reduce the impact of other factors, this study includes the following control variables based on prior literature [40,41]: agricultural development level, which is a crucial underpinning for rural industrial development and reflects the regional agricultural development. The ratio of added value of the primary industry to the total output value of the region and logarithmic transformation (lnadl) have been employed to measure it. Rural residents' standard of living should encompass not only their income level but also advances in their consumption capacity in numerous aspects. Thus, this study has utilized the Engel coefficient of rural occupants and logarithmic transformation (lnfls) to evaluate it. Financial backing from local governments is pivotal to economic development. For measurement, this study has employed the expenditure of local financial affairs related to agriculture, forestry, and water conservation and logarithmic transformation (lnfs). Urbanization advancement can facilitate the movement of resources to rural areas, driving the rejuvenation and amplification of rural industries. The proportion of urban population to rural population and logarithmic transformation (lnul) were utilized to gauge this aspect. Please consult Table 1 for detailed definitions and assignments of variables.

**Table 1:** Definition and assignment of model variables.

Variable Category	Variable Name	Variable Symbol	Variable Assignment
Dependent Variable	Development Level of Rural Industries	ridli	Index of Development Level of Rural Industries
Core Independent Variable	Average Agricultural Insurance Payment per mu	L.acp	Lagged one period of (Agricultural Insurance Payment per mu/Grain Sowing Area)
Control Variables	Level of Agricultural Development	lnadl	Natural logarithm of ratio of value-added of primary sector to regional GDP
	Standard of Living of Farmers	lnfls	Natural logarithm of Engel coefficient for rural residents
	Level of Financial Support	lnfs	Natural logarithm of government expenditures on agriculture, forestry, and water resources at the local level
	Level of Urbanization	lnul	Natural logarithm of ratio of urban population to rural

## 5 5Empirical analysis

### 5.1 Calculation of rural industry development index

To provide a comprehensive and accurate representation of the dependent variable, i.e., the level of rural industry development, this study has constructed an index system to compute the rural industry development index. The index system has been established from the perspective of the primary, secondary, and tertiary industries, based on relevant documents on rural revitalization and rural industry development released by the Chinese central government. It includes six indicators (listed in Table 2): the per capita output value of agriculture, forestry, animal husbandry, and fishery; the total power of agricultural machinery per unit area; investment in fixed assets for personal services and other service industries by rural residents; intensity of fertilizer application; intensity of pesticide application, and employment in rural private enterprises.

**Table 2:** Index system for evaluating rural industry development level.

Target Level	Indicator Name	Indicator Source	Variable Symbol
Evaluation of Rural Industry Development Level	Per Capita Agriculture, Forestry, Animal Husbandry, and Fishery GDP	Regional Agriculture, Forestry, Animal Husbandry, and Fishery GDP/Rural Population	x1
	Total Power of Agricultural Machinery per Unit Area	Total Power of Agricultural Machinery/Crop Planting Area	x2
	Personal Fixed Investment of Rural Residents in Service and Other Service Industries (in 100 million yuan)	Directly Obtained	x3
	Intensity of Fertilizer Application	Pure Amount of Fertilizer Application per Unit Area/Crop Planting Area	x4
	Intensity of Pesticide Application	Amount of Pesticide Application/Crop Planting Area	x5
	Number of Employees in Rural Private Enterprises	Directly Obtained	x6

To determine the score of each indicator for each year, the panel entropy method has been utilized. Firstly, the weight of each indicator is calculated (as demonstrated in Table 3), and subsequently, the rural industry development index for each province from 2011 to 2019 is computed (as shown in Table 4).

**Table 3:** Index weight (2011–2019).

Year	x1	x2	x3	x4	x5	x6
2011	0.0984	0.1417	0.3842	0.2811	0.0700	0.0246
2012	0.1218	0.1524	0.3661	0.2693	0.0595	0.0310
2013	0.1211	0.1462	0.4049	0.2466	0.0565	0.0245
2014	0.1148	0.1286	0.4579	0.2295	0.0446	0.0245
2015	0.1068	0.1284	0.4573	0.2391	0.0437	0.0247
2016	0.1068	0.1611	0.4042	0.2516	0.0452	0.0311
2017	0.1034	0.1822	0.3779	0.2480	0.0601	0.0284
2018	0.1069	0.1791	0.3526	0.2468	0.0643	0.0503
2019	0.0999	0.1847	0.3726	0.2533	0.0701	0.0195

**Table 4:** Scores of rural industry revitalization and development level in various provinces and regions (2011–2019).

Province	2011	2012	2013	2014	2015	2016	2017	2018	2019
Beijing	0.2658	0.2622	0.2205	0.2224	0.2369	0.2747	0.3022	0.3023	0.2822
Tianjin	0.2163	0.2382	0.2150	0.1943	0.1965	0.2293	0.2522	0.2223	0.1688
Hebei	0.4216	0.3989	0.3592	0.2412	0.2557	0.2447	0.2658	0.3021	0.4078
Shanxi	0.1996	0.2086	0.2182	0.2101	0.2160	0.1846	0.1911	0.2117	0.1798
Inner Mongolia	0.2264	0.2322	0.2327	0.1937	0.2157	0.1845	0.1940	0.2279	0.2068
Jilin	0.2239	0.2516	0.1609	0.1355	0.1347	0.1475	0.1557	0.1735	0.1527
Heilongjiang	0.2479	0.2889	0.2180	0.1944	0.1736	0.1752	0.2089	0.2175	0.1914
Jiangsu	0.8160	0.8143	0.4424	0.3918	0.4023	0.3912	0.4679	0.5014	0.4034
Zhejiang	0.4121	0.4336	0.4213	0.3748	0.4176	0.6774	0.4777	0.3530	0.4344
Anhui	0.1929	0.2020	0.2014	0.1951	0.1591	0.2890	0.2207	0.4164	0.2341
Fujian	0.1540	0.1837	0.1802	0.1784	0.2216	0.2478	0.2641	0.2622	0.2216
Jiangxi	0.3365	0.3494	0.1730	0.1601	0.2175	0.1507	0.1555	0.1723	0.1581
Shandong	0.4025	0.4448	0.4789	0.5324	0.5872	0.6769	0.7555	0.7787	0.7445
Henan	0.3650	0.3863	0.6039	0.6410	0.5492	0.2207	0.2431	0.4760	0.2821
Hubei	0.1593	0.2368	0.2230	0.1992	0.2388	0.2964	0.2602	0.4300	0.3023
Hunan	0.3000	0.3180	0.2689	0.1790	0.1848	0.3544	0.4014	0.3654	0.3732
Guangdong	0.3465	0.2300	0.2756	0.1729	0.1971	0.1882	0.2007	0.1810	0.1890
Guangxi	0.2050	0.3172	0.5018	0.5395	0.6321	0.5708	0.5606	0.5474	0.5487
Hainan	0.1417	0.1815	0.1745	0.1605	0.1581	0.1934	0.2175	0.2021	0.2322
Chongqing	0.1312	0.1395	0.1400	0.1373	0.1463	0.1616	0.1555	0.1762	0.1487
Sichuan	0.2136	0.2241	0.2394	0.2479	0.1566	0.1596	0.4172	0.1564	0.1460
Guizhou	0.1240	0.1412	0.1362	0.1464	0.1685	0.1786	0.1885	0.2721	0.1938
Yunnan	0.1518	0.1637	0.1374	0.1262	0.2005	0.2542	0.1712	0.1520	0.2477
Shaanxi	0.1727	0.2171	0.1381	0.1405	0.1300	0.1366	0.1727	0.1931	0.1384
Gansu	0.1935	0.1640	0.2067	0.2005	0.2116	0.2407	0.2997	0.3576	0.2395
Qinghai	0.1817	0.1851	0.1648	0.1506	0.1424	0.1908	0.2186	0.2422	0.2162
Ningxia	0.1530	0.1675	0.1566	0.1417	0.1450	0.1305	0.1457	0.1676	0.1471
Xinjiang	0.1331	0.1589	0.1683	0.1474	0.1330	0.1555	0.2021	0.2429	0.1979

### 5.2 Benchmark regression

Using a fixed-effects model, this paper examines the effect of agricultural insurance on rural industrial development. To estimate the impact, we conducted regression analyses separately for region-fixed and region-time fixed models. We examined the core explanatory variables and evaluated their impact in the presence and absence of control variables (shown in Table 5). Table 5(1) and (2) present the results of agricultural insurance (one-period lagged per-mu agricultural insurance claims payment expenditure) on rural industrial development under the region-fixed and two-way fixed models, respectively. Table 5(3) and (4) further demonstrate that the core explanatory variable remains significantly positive even after the inclusion of control variables in the region-fixed and two-way fixed models, respectively, indicating a positive impact of agricultural insurance on rural industrial development.

**Table 5:** Panel model regression results.

Variable Symbol	（1）	（2）	（3）	（4）
L.acp	0.1591*** (0.046)	0.1648*** (0.0421)	0.0119** (0.0070)	0.1700*** (0.0399)
logadl	–	–	0.0040 (0.1273)	0.0317 (0.0589)
logfls	–	–	0.5377** (0.1850)	−0.3750** (0.2007)
logfs	–	–	0.0370 (0.1142)	0.2499*** (0.0478)
logul	–	–	0.5176* (0.3115)	0.1232 (0.1029)
Regional Fixed	Yes	Yes	Yes	Yes
Time Fixed	No	Yes	No	Yes
_cons	0.2257*** (0.0120)	0.2113*** (0.0309)	−0.7292* (0.4373)	−0.0072* (0.3533)
R-sq	0.0813	0.0967	0.0592	0.2563

Note: Standard errors in parentheses are heteroscedasticity-robust; *, **, and *** indicate statistical significance at the 10 %, 5 %, and 1 % levels, respectively.

Based on the empirical findings from the two-way fixed effects model, Table 5(2) demonstrates that agricultural insurance development significantly and positively affects rural industrial revitalization at a 5 % level of significance, even without the inclusion of control variables. Furthermore, after incorporating control variables, Table 5(4) confirms the robustness of the estimation results, showing that agricultural insurance development continues to have a significant positive effect on rural industrial development at a 1 % level of significance. Moreover, the inclusion of control variables enhances the model fit, as evidenced by the increase in goodness of fit (R-sq) from 0.0967 to 0.2563.

The estimation results from the region-fixed effects model indicate that farmers' living standards have a significant negative impact on rural industrial development. A decrease of 1 % in Engel's coefficient of rural residents corresponds to a 0.5377 unit increase in the rural industrial development index. Conversely, urbanization has a significantly positive impact on rural industrial development, with an increase of 1 % in the urban-to-rural population ratio resulting in a 0.5176 unit increase in the rural industrial development index. The remaining control variables are not found to be statistically significant.

In contrast, the estimation results from the two-way fixed effects model reveal that the impact of farmers' living standards on rural industrial development remains significantly negative. A decrease of 1 % in Engel's coefficient of rural residents corresponds to a 0.3750 unit increase in the rural industrial development index, which differs from the results obtained in the region-fixed effects model. Additionally, the impact of financial support on rural industrial development is significantly positive, with an increase of 1 % in local government agricultural and forestry water affairs expenditure leading to a 0.2499 unit increase in the rural industrial development index. No significant effects were observed for the other control variables.

### 5.3 Analysis of regional heterogeneity

This paper classifies provinces and regions into two groups: the first group consists of seven major grain-producing provinces--Heilongjiang, Henan, Shandong, Jilin, Hebei, Inner Mongolia, and Liaoning--while the remaining 22 provinces and regions are categorized as non-major grain-producing provinces. As shown in Table 6(1) and (2), the estimation results from major grain-producing provinces indicate insignificant effects for the core explanatory variables, both before and after controlling for other variables, with coefficients that do not exhibit negative values. One possible explanation for the lack of significance in major grain-producing provinces could be attributed to China's current reliance on policy-based agricultural insurance, which is subsidized by the central and local governments. However, financially-constrained major grain-producing provinces often face challenges in providing adequate support for agricultural insurance. Additionally, insurance companies operating in these areas may display less enthusiasm towards agricultural insurance operations due to the uncertainties and frequency of agricultural risks.

**Table 6:** Regional regression results.

Variable Symbol	Major grain-producing provinces		Non-grain-producing provinces	
	（1）	（2）	（3）	（4）
L.acp	−0.5407 (0.4313)	−0.2421 (0.2828)	0.2921*** (0.03970)	0.2525*** (0.03931)
logadl	–	−1.1569 (1.2891)	–	0.2556** (0.1155)
logfls	–	−7.3612* (4.1665)	–	−0.3811* (0.1987)
logfs	–	0.2400 （0.1636）	–	0.1370* (0.0704)
logul	–	−0.5145 0.8240	–	0.1220 (0.1875)
Regional Fixed	Yes	Yes	Yes	Yes
Time Fixed	Yes	Yes	Yes	Yes
_cons	0.4151** (0.1543)	11.6850 （7.1260）	0.1312*** (0.0376)	−0.0072* (0.3533)
R-sq	0.3267	0.9465	0.7079	0.8243

Note: Standard errors in parentheses are heteroscedasticity-robust; *, **, and *** indicate statistical significance at the 10 %, 5 %, and 1 % levels, respectively.

In addition, China's agricultural insurance working principle of “low protection and wide coverage” imposes limitations on the level of agricultural insurance protection, as it fails to adequately cover material costs, resulting in low participation rates among farmers. Furthermore, it is important to note that the study period is limited, which means that recent innovations in fully cost-based insurance and planting income insurance, introduced in 2019 for three major grain crops and aiming to encompass all major grain-producing counties in the 13 major grain-producing provinces by 2021, were not included in the research conducted for this paper. These factors, collectively, contribute to the insignificant results observed in the study.

Based on Table 6(3) and (4), the estimation results from non-major grain-producing provinces reveal that the core explanatory variable, the development level of agricultural insurance (one-period lag of per mu agricultural insurance claims expenditure), is significantly positive at the 1 % level before controlling for other variables. This finding indicates that the development of agricultural insurance has a substantial positive impact on rural industry development.

### 5.4 Robustness check

In order to ensure the reliability and stability of the empirical results, this study follows the established practice of the academic community [42,43] and employs the method of variable replacement to conduct robustness checks. Table 7 presents the results of these checks, where the index of rural industrial development level is replaced with the added value of the primary industry and the variable measuring agricultural insurance development level, initially measured by per mu agricultural insurance claim payment expenditure, is replaced with the regional agricultural insurance premium income variable. The estimation results of the regional fixed effects model in the first column of Table 7 indicate that the substituted variable of rural industrial development level is significantly and positively associated with the added value of the primary industry. Specifically, when the regional agricultural insurance premium income increases by 1 %, the added value of the primary industry increases by 0.181 %. Similarly, the estimation results of the two-way fixed effects model in the second column of Table 7 demonstrate that the replacement variable of rural industrial development level is significantly and positively related to the added value of the primary industry. When the regional agricultural insurance premium income increases by 1 %, the added value of the primary industry increases by 0.546 %. These robustness checks provide additional evidence that the influence of agricultural insurance on rural industrial development is robust.

**Table 7:** Robustness test results.

Variable Name	Variable Description	Variable Symbol	（1）	（2）
Dependent Variable	Logarithm of the added value of primary industry	lnavpi	–	–
Core Explanatory Variable	Logarithm of agricultural insurance premium income by region	lnadi	0.1810*** （0.0245）	0.5464*** （0.04582）
		_cons	2.6388*** (0.0720)	1.7909*** (0.1215)
Regional Fixed			Yes	Yes
Time Fixed			No	Yes
R-sq			0.5769	0.5160

Note: Standard errors in parentheses are heteroscedasticity-robust; *** indicates statistical significance at the 1 % level.

## 6 Discussion and practical applications

### 6.1 Discussion

(1) The empirical results indicate that agricultural insurance positively influences the development of rural industry, consistent with the anticipated research hypothesis H1a. This suggests that the progression of agricultural insurance can significantly bolster rural industrial development. While existing literature has predominantly focused on specific aspects of the agricultural industry or production process, conclusions drawn from these studies highlight the notable role of agricultural insurance. For instance, agricultural insurance affects farmers' adoption of new technologies [44] and their investment in agricultural production inputs [45], underscoring its significance in agricultural production.

Conversely, this study's findings reveal that the influence of agricultural insurance extends beyond isolated sectors, potentially affecting the rural industry as a whole. This broader impact highlights the significance of agricultural insurance in shaping the overall landscape of rural economic development.These empirical outcomes further expand our understanding of the extensive functionalities of agricultural insurance. Unlike previous studies that concentrated on the influence of agricultural insurance on specific components or the overall agricultural industry, our study demonstrates its positive impact on rural industry as a whole, offering a distinct yet complementary perspective.

Moreover, numerous scholars have investigated the role of rural finance [40,41] and agricultural credit in the development of rural industries [46], concluding that rural finance is crucial for rural industrial growth. Our empirical findings corroborate this view, demonstrating that agricultural insurance, as an integral part of rural finance, plays a vital role in advancing rural industry development.

(2) Further research has revealed the heterogeneous impact of agricultural insurance on the development of rural industries, aligning with our anticipated hypothesis H2b. In provinces characterized as major grain producers, the influence of agricultural insurance on rural industry development is not pronounced. Conversely, in provinces where grain production is less prominent, agricultural insurance significantly fosters the growth of rural industries. Many scholars have acknowledged that agricultural insurance's effects vary regionally, influenced primarily by climatic conditions, agricultural production characteristics, policy, and other region-specific factors [47,48].

Specifically, in major grain-producing provinces, the impact of agricultural insurance on rural industry development is not significant, corroborating findings from existing literature. For instance, Jiang et al. [49] investigated the relationship between agricultural insurance coverage and food security across various grain production and distribution areas. Their empirical analysis revealed that in major grain production and distribution zones, agricultural insurance coverage does not significantly influence food security. Similarly, Nie et al. [50] observed a positive impact of agricultural insurance on grain production in regions that are not major grain producers, whereas no significant effect was noted in the major grain-producing districts. This observation is consistent with our findings, where in major grain-producing provinces, the effect of agricultural insurance on rural industry is negligible, while in non-major grain-producing provinces, the effect is pronounced. So, what accounts for the lack of significant impact of agricultural insurance in major grain-producing areas? This study posits that the reason may lie in the underdeveloped nature of agricultural insurance in these regions. These provinces often suffer from limited financial resources and lack substantial local government support for agricultural insurance. Moreover, the agricultural insurance products available may not provide sufficient coverage, impeding the development of agricultural insurance and consequently, the growth of rural industries. However, Xu et al. [51] found that agricultural insurance positively influences agricultural production in China's major grain-producing regions. This difference in findings could be due to the divergent regional focus of the studies. Hence, the effectiveness of agricultural insurance in major grain-producing provinces compared to other provinces requires further exploration in future research.

### 6.2 Practical applications

Building on the discussions within this paper, we propose the following recommendations aimed at providing insights for the optimization of agricultural insurance policies in China, and serving as a reference for insurance companies engaging in agricultural insurance operations. This is intended to contribute to a more effective framework for supporting and protecting the agricultural sector within the context of evolving risks and challenges.

(1) Diversify agricultural insurance products to cater to the varied planting requirements of farmers. Insurance companies can develop localized insurance products that align with regional attributes, such as insurance for miscellaneous grains and beans in Heilongjiang Province and large yellow croaker price index insurance in Fujian Province. These initiatives provide farmers with effective measures to mitigate agricultural risks and support regional agricultural development.
(2) Incrementally increase the extent of agricultural insurance coverage and expand the range of premium subsidies. The central government should offer certain premium subsidies for essential agricultural and livestock varieties, while major grain-producing provinces should receive policy incentives and marginally higher premium subsidies. Provinces should provide specific premium subsidies for unique agricultural commodities to support localized agricultural operations that align with their respective orientations and development strategies.
(3) Enhance the interaction between banks and insurance companies and devise new “agricultural insurance + credit” models. To address funding issues faced by farmers, agricultural insurance and banking-oriented financial institutions should develop effective collaborations. Furthermore, exploring more approaches and content for the utilization of agricultural insurance funds can maximize its role in rural revitalization and development.

## 7 Conclusion and future prospects

### 7.1 Conclusion

Rural industry is widely recognized as a cornerstone for rural revitalization, and agricultural insurance holds a pivotal role in rural finance. This study aims to examine the influence of agricultural insurance on the development of rural industry. The paper adopts a theoretical approach that utilizes the principle of functional decomposition to dissect the functions of agricultural insurance and analyze its impact on the development of rural industry. Based on this theoretical framework, a research hypothesis is proposed, which serves as the foundation for empirical testing using the fixed effect model. Through empirical analysis, this study explores the empirical relationship between agricultural insurance and the development of rural industry. Based on the research presented above, this paper concludes that agricultural insurance exerts a positive influence on the development of rural industries. Furthermore, the impact of agricultural insurance on the advancement of rural industries is characterized by heterogeneity. This conclusion underscores the nuanced role of agricultural insurance as a facilitator of rural economic growth, highlighting its varied effects across different sectors within the rural economy.

### 7.2 Limitations and future prospects

Acknowledging the limitations in our knowledge and data, this research unavoidably carries certain biases. To mitigate these limitations, we have devised a plan to conduct field surveys that will supplement our research and provide clearer insights into the issue at hand. These surveys will enable us to gather first-hand data and validate the findings of our study.

Furthermore, we intend to delve into related research areas to further enhance the comprehensiveness of our study. In the next phase of our research, we will explore the impact of agricultural insurance on rural enterprises across various scales and industries. This expanded scope will allow us to capture the broader effects of agricultural insurance on rural development and industry growth.

By incorporating field surveys and expanding our research scope, we aim to strengthen the validity and reliability of our findings, offering a more comprehensive understanding of the relationship between agricultural insurance and rural development.

## Ethics approval and consent to participate

Not applicable.

## Funding

This research was funded by 10.13039/501100007314Minnan Normal University President's Fund Project (Grant No. SK18017), Fujian Provincial Social Science Foundation Project (Grant No. FJ2022BF026), Fujian Innovation Strategy Research Project (Grant No. 2023R0055), 10.13039/501100012456National Social Science Foundation of China (Grant No. 23XJY011), Major Project of Basic Theoretical Research Base of Philosophy and Social Sciences under the Guidance of Marxism in Fujian Province (Grant No. FJ2024MGCA022).

## Data availability statement

The datasets analyzed during the current study are available from the corresponding author on reasonable request. The data used in this study primarily derives from the ‘China Statistical Yearbook’, ‘China Insurance Yearbook’, and ‘China Rural Statistical Yearbook’ (2010–2020). For publicly available data sources, corresponding online links are provided. Detailed page references and access information for all data can be found in the appendix.

## CRediT authorship contribution statement

**Dainan Hou:** Writing – original draft, Funding acquisition, Formal analysis, Data curation, Conceptualization. **Xin Wang:** Writing – review & editing, Formal analysis, Data curation, Conceptualization.

## Declaration of competing interest

The authors declare that they have no known competing financial interests or personal relationships that could have appeared to influence the work reported in this paper.

## Figures and Tables

**Table 1:** Data Description for Calculating the Index of Rural Industry Revitalization and Development Level

Indicator Name	Source of Indicator	Variable Symbol	The source of the indicators comes from the yearbooks∗
Per Capita Agriculture, Forestry, Animal Husbandry, and Fishery GDP	Regional Agriculture, Forestry, Animal Husbandry, and Fishery GDP/Rural Population	x1	The data on the regional GDP of agriculture, forestry, animal husbandry, and fishery are derived from the “China Statistical Yearbook” (2010–2020). These data can be found in the online version of the China Statistical Yearbook at https://www.stats.gov.cn/sj/ndsj/. Specifically, the data from 2010 to 2013 are included in “Tables 13–4" of each year's yearbook, while the data from 2014 to 2020 are in “Tables 12–3" of each respective yearbook. The data on the rural population come from the “China Statistical Yearbook” (2010–2020). These data can also be found in the online version of the yearbook at https://www.stats.gov.cn/sj/ndsj/. For each specific year, the data from 2010 to 2012 are included in “Tables 3–8" of each year's yearbook, the 2013 data in “Tables 3–7", the data for 2014 to 2015 in “Tables 2–7", and the data from 2016 to 2020 in “Tables 2–8".
Total Power of Agricultural Machinery per Unit Area	Total Power of Agricultural Machinery/Crop Planting Area	x2	The data on the total power of agricultural machinery are sourced from the “China Statistical Yearbook” (2010–2020). These can be found in the online edition of the yearbook at https://www.stats.gov.cn/sj/ndsj/. Specifically, the data from 2010 to 2013 are recorded in “Tables 13–5" of each year's yearbook, and from 2014 to 2020, the data are in “Tables 12–4". The data on the sown area of crops are sourced from the “China Statistical Yearbook” (2010–2020). These data can be found in the online version of the yearbook at https://www.stats.gov.cn/sj/ndsj/. Specifically, the data from 2010 to 2013 are included in “Tables 13–13" of each year's yearbook, while the data from 2014 to 2020 are in “Tables 12–9" of each respective yearbook.
Personal Fixed Investment of Rural Residents in Service and Other Service Industries (in 100 million yuan)	Directly Obtained	x3	This data comes from the “China Rural Statistical Yearbook” (2010–2020), specifically located in the fifth section “Rural Investment” of the yearbook, under “Tables 5–2".
Intensity of Fertilizer Application	Pure Amount of Fertilizer Application per Unit Area/Crop Planting Area	x4	The data on the application of chemical fertilizers in terms of pure nutrient are from the “China Statistical Yearbook”. Specifically, the data from 2010 to 2013 can be found in “Tables 13–6" of the respective year's yearbook, while the data from 2014 to 2020 are included in “Tables 12–5" of each year's yearbook. The data on the sown area of crops are from the “China Statistical Yearbook” (2010–2020). For this data, the information from 2010 to 2013 is located in “Tables 13–13" of each year's yearbook, while the data from 2014 to 2020 are in “Tables 12–9".
Intensity of Pesticide Application	Amount of Pesticide Application/Crop Planting Area	x5	The data on the usage of pesticides are from the “China Rural Statistical Yearbook”, specifically included in the third section “Rural Basic Conditions and Agricultural Production Conditions” under “Tables 3–9". The data on the sown area of crops come from the “China Statistical Yearbook” (2010–2020). Specifically, the data from 2010 to 2013 are located in “Tables 13–13" of each year's edition, while the data from 2014 to 2020 are in “Tables 12–9" of each respective year's edition.
Number of Employees in Rural Private Enterprises	Directly Obtained	x6	This data is from the “China Statistical Yearbook”, specifically found in “Tables 4–8".

∗ Note: The years mentioned in this column refer to the publication year of the yearbook, and the corresponding data are all from the previous year.

**Table 2:** Data Sources for Variables Used in Empirical Analysis

Variable Name	Variable Symbol	Variable Assignment	The source of the indicators comes from the yearbooks∗
Development Level of Rural Industries	ridli	Index of Development Level of Rural Industries	The data on agricultural insurance claim payments come from the local editions of the “China Insurance Yearbook,” specifically from the “Business Statistics Table of Each Property Insurance Branch Company” in each province and region. The data on the sown area of grains are sourced from the “China Statistical Yearbook”. Specifically, the data from 2010 to 2013 can be found in “Tables 13–13" of the respective year's yearbook, while the data from 2014 to 2020 are included in “Tables 12–9" of each year's yearbook.
Average Agricultural Insurance Payment per mu	L.acp	Lagged one period of (Agricultural Insurance Payment per mu/Grain Sowing Area)	The indicator of the proportion of the primary industry's added value to the regional gross production value (%) can be found in the “China Rural Statistical Yearbook”, specifically in the second section “Comprehensive and Summary” under “Tables 2–4 The Status of Rural Economy in National Economy by Region".
Level of Agricultural Development	lnadl	Natural logarithm of ratio of value-added of primary sector to regional GDP	The data on the Engel coefficient of rural residents come from the “China Statistical Yearbook”. The location of the data for different years is as follows: The data for 2010, 2011, and 2012 are in “Tables 10–26" calculated by combining food consumption with total living expenses. The 2013 data are in “Tables 11–24". The 2014 data are included in “Tables 6–30". The 2015 data are in “Tables 6–28". The data from 2016 to 2020 are in “Tables 6–32".
Standard of Living of Farmers	lnfls	Natural logarithm of Engel coefficient for rural residents	The data on local government expenditures for agriculture, forestry, and water affairs come from the “China Statistical Yearbook”. Specifically, the location of the data for each year is as follows: The data for 2010, 2011, and 2012 are in “Tables 8–8". The 2013 data are included in “Tables 9–6". The data from 2014 to 2020 are in “Tables 7–6".
Level of Financial Support	lnfs	Natural logarithm of government expenditures on agriculture, forestry, and water resources at the local level	The 2010 data are from “Tables 3–4" of the “China Statistical Yearbook". For 2011, due to the absence of urban population data in the “China Statistical Yearbook,” the urban population was calculated by subtracting the rural population from the total population at year-end in each region as listed in “Tables 3–13". The rural population data for 2011 come from the “China Rural Statistical Yearbook,” specifically from “Tables 3–2 Population and Employed Persons in Rural Areas by Region” in the third section “Rural Basic Conditions and Agricultural Production Conditions". The 2012 data are from “Tables 3–5" of the “China Statistical Yearbook". The 2013 data are from “Tables 3–7" of the “China Statistical Yearbook". The data for 2014 to 2015 are included in “Tables 2–7" of the “China Statistical Yearbook". The data for 2016 to 2020 are in “Tables 2–8" of the “China Statistical Yearbook".

∗ Note: The years mentioned in this column refer to the publication year of the yearbook, and the corresponding data are all from the previous year.

## Appendix

This study utilized three types of yearbooks: the “China Statistical Yearbook,” the “China Rural Statistical Yearbook,” and the “China Insurance Yearbook,” covering the period from 2010 to 2020. Each yearbook is published annually and contains data from the previous year. For instance, data from 2009 are included in the 2010 edition of the yearbook. The electronic version of the “China Statistical Yearbook” can be found on the official website of the National Bureau of Statistics of China (https://www.stats.gov.cn/sj/ndsj/). The specific data indicators used in this study and their sources are detailed in tables; please refer to Table 1, Table 2 for more information.
